# A Machine Learning Model Based on GRU and LSTM to Predict the Environmental Parameters in a Layer House, Taking CO_2_ Concentration as an Example

**DOI:** 10.3390/s24010244

**Published:** 2023-12-31

**Authors:** Xiaoyang Chen, Lijia Yang, Hao Xue, Lihua Li, Yao Yu

**Affiliations:** 1 College of Mechanical and Electrical Engineering, Hebei Agricultural University, Baoding 071001, China; 2 Key Laboratory of Meat and Layer Breeding Facilities Engineering, Hebei Agricultural University, Baoding 071001, China; 3 Hebei Provincial Key Laboratory of Livestock and Poultry Breeding Intelligent Equipment and New Energy Utilization, Hebei Agricultural University, Baoding 071001, China; 4 Research Centre of Information Technology, Beijing Academy of Agriculture and Forestry Sciences, Beijing 100097, China

**Keywords:** layer house, CO_2_ concentration, prediction, GRU, LSTM

## Abstract

In a layer house, the CO_2_ (carbon dioxide) concentration above the upper limit can cause the oxygen concentration to be below the lower limit suitable for poultry. This leads to chronic CO_2_ poisoning in layers, which manifests as listlessness, reduced appetite, weak constitution, decreased production performance, and weakened resistance to disease. Regulating ventilation may ensure a suitable CO_2_ concentration in layer houses. Predicting the changes in CO_2_ concentration and regulating the CO_2_ concentration in advance are key to ensuring healthy large-scale breeding of layers. In recent years, machine learning and deep learning methods have been increasingly applied to this field. A CO_2_ prediction model for layer house is proposed based on a GRU (gated recurrent unit) and LSTM (long short-term memory). The temperature, humidity, and CO_2_ were determined as inputs to the model by the correlation coefficient. The datasets of the experimental layer house were continuously measured during June–July 2023, using a self-developed environmental monitor, and the monitored data were used as samples for model inputs. There were 22,000 time series data in the datasets. In this study, multivariate time series data were standardized via data pre-processing to improve model training. GRU and LSTM models were constructed. The models were trained using a training set. Then, these trained models were used to provide predictions on a test set. The prediction errors were calculated using the true values of the test set and the predicted values provided by the models. To test the performance of the model and accuracy of the predictions, predictions were made for different numbers of datasets. The results demonstrated that the combined prediction model had good generalization, stability, and convergence with high prediction accuracy. Due to the structure of the model, the stability of the LSTM model was higher than that of the GRU model, and its prediction accuracy and speed were lower than those of the GRU model. When the datasets of the GRU model were 15,000 to 17,000, The MAE of the GRU was 70.8077 to 126.7029 ppm, and the prediction time of the GRU is 16 to 24 ms. When the LSTM model’s datasets were 15,000–20,000, the MAE of LSTM was 78.8596 to 136.0896 ppm, and the prediction time of the GRU was 17 to 26 ms.

## 1. Introduction

A closed layer house with stacked cages is a relatively closed environment. Thus, the environmental factors in the house, such as temperature, relative humidity, and harmful gases and dust concentrations, can substantially impact the growth, development, reproduction, production, and health of the poultry [1]. The harmful gases produced in a layer house not only pollute the environment but also affect the physical health of layers or suppress their production potential, resulting in economic loss in the farming industry. These harmful gases mainly include NH_3_ (ammonia), H_2_S (hydrogen sulfide), and CO_2_, which can be produced at harmful concentrations. Some of these harmful gases do not have a distinct odor and do not pose any serious hazards for short exposures and when at low concentrations. However, some gases damage health at high concentrations and result in acute symptoms, but this is less likely to occur under poultry production conditions. Long-term exposure to low concentrations of harmful gases causes progressive harm to both human staff and poultry health and reduces poultry productivity, causing the health status of the poultry to be poor, resulting in reduced immunity and reduced production performance. The symptoms of this condition can be easily confused with those of malnutrition or invasion by pathogenic microorganisms. Consequently, determining whether the poultry condition is due to the effects of long-term exposure to low concentrations of harmful gases can be difficult. Therefore, in modern farming and production, the air quality problem in livestock housing must be addressed [2]. In [3], it is reported that CO_2_ emission linearly increases with increasing layer age in days during broiler farming and production. Higher concentrations of NH_3_ and CO_2_ in animal barns can negatively affect production and health of animals and workers. Additionally, indoor and ambient environmental conditions such as temperature and relative humidity were measured simultaneously with pollutant gas concentrations [4].

Pollutants emitted from poultry housing facilities are a concern from human health, bird welfare, and environmental perspectives. Development of emission factors for these aerial pollutants is difficult due to variable climatic conditions, the number and type of poultry, and the wide range of management practices used [5].

In recent years, machine learning and deep learning methods have been increasingly applied to this field.

At present, prediction models for time series include SVM (support vector machine), ARIMA (autoregressive integrated moving average), and RF (random forest). The computational complexity of the SVM algorithm is relatively high, especially for large-scale and high-dimensional datasets, which require a large amount of computation time and space. In addition, the training process of the SVM algorithm requires multiple iterations, which also increases the computational complexity. The RF algorithm is more complex, computationally expensive, and requires more time to train than other algorithms [6]. The ARIMA model requires complete time series data with no missing values. In practical applications, there are often missing and incomplete data, which affects the effectiveness of the model [7,8].

The prediction method based on recurrent neural networks has advantages in terms of prediction accuracy and speed. In [9], a novel PIHNN (physics-informed hybrid neural network) model based on the LSTM neural network is proposed. In [10], Chinese stock returns were modeled and predicted using LSTM. The historical data of the Chinese stock market were transformed into 30-day-long sequences with 10 learning features and 3-day earning rate labeling. The model was fitted by training on 900,000 sequences and tested using another 311,361 sequences. In [11], a CNN (convolutional neural network)–LSTM model is proposed to predict gas field production based on a gas field in southwest China. The CNN has a feature extraction ability, and LSTM can learn sequence dependence. By the combination of the two abilities, the CNN–LSTM model can describe the changing trend of gas field production. In [12], augmentation of fully convolutional networks with LSTM–RNN (recurrent neural) submodules for time series classification is proposed. The proposed LSTM–FCN (fully convolutional network) achieves state-of-the-art performance compared with others. In [13], a novel traffic forecast model is proposed based on an LSTM network. Differently from conventional forecast models, the proposed LSTM network considers temporal–spatial correlation in a traffic system via a two-dimensional network that is composed of many memory units. A comparison with other representative forecast models validated that the proposed LSTM network can achieve better performance. In [14], a GRU network is proposed based on experience replay and the Snake Optimizer for real-time prediction in real-world non-stationary channels.

At present, there are relatively few studies on the prediction of carbon dioxide concentration in small layer houses in China. In addition, most existing studies rely on single-model prediction.

In this study, LSTM and GRU models with good prediction performance were constructed to predict the carbon dioxide concentration in a small layer house.

The second part of the paper introduces the acquisition of the datasets, the research of GRU and LSTM networks, and the construction of the prediction models. The third part draws the curve between the predicted and actual values of the model, draws the MAE and MSLE curves of the model prediction, and analyzes the performance of the GRU and LSTM models. The fourth part discusses the research of the paper. The fifth part is the conclusion of the paper.

## 2. Materials and Methods

### 2.1. Data Acquisition

**Figure 1:**
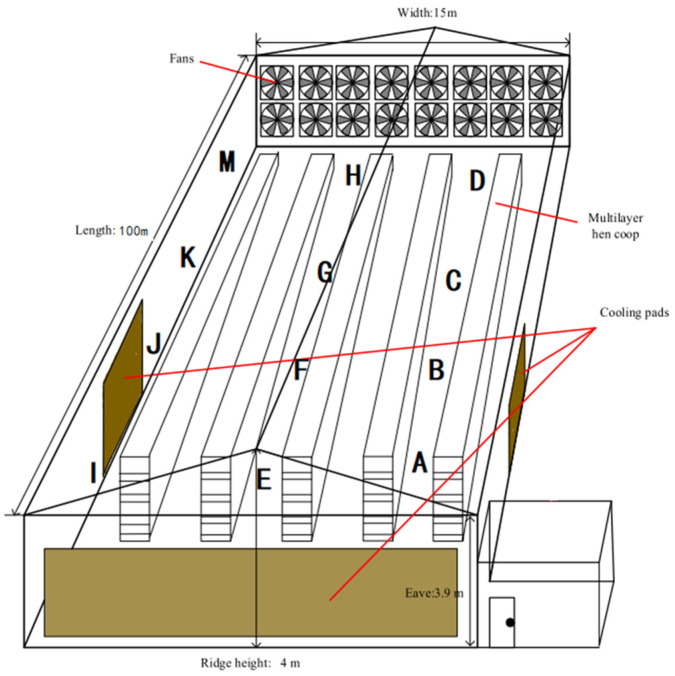
The on-site image of the measurement experiment in the layer house.

**Figure 2:**
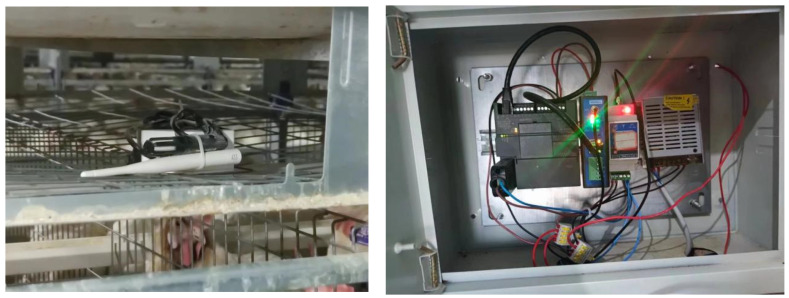
The temperature monitoring device in the laying hen house. The measurements lasted from May to June 2023.

The experiment was conducted in a layer house from January to December in 2023. The selected experimental layer house was an intensive closed layer house. Each layer house was 100 m long, 15 m wide, and 4 m high, with five rows and four layers of cage equipment. The breed of the layers was Hailan grey, with a feeding density of 74 layers per group, and the designed feeding capacity of a single building was 53,280 layers. The layer house utilized a combined positive and negative pressure ventilation system with a longitudinal design. The positive pressure ventilation system consisted of 95 fans with a diameter of 300 cm each, and the negative pressure ventilation system consisted of 18 fans with a diameter of 1400 cm each. The house was equipped with wet curtains, covering a total area of 119 m^2^. The monitoring device consisted of a data acquisition node, master control module, and remote terminal management platform and continuously collected data from 00:00 to 24:00 every day. There were 12 devices in the layer house, from A to M. The laying hen house and the monitoring device in the layer house are shown in Figure 1 and Figure 2.

### 2.2. Correlation Coefficient

The layer house CO_2_ concentration exhibits time-series patterns and autocorrelation, that is, the data from the previous moment influences the current change of data [15]. Therefore, a model should not only consider the influence of real-time environmental factors but also the influence of historical data on the current moment [16].

To improve the effectiveness of the model input information and reduce irrelevant noise interference, this study adopted the correlation coefficient to analyze the degree of influence of the factors on the layer house CO_2_ concentration and selected the factors with high correlation as the model’s inputs [17]. The correlation coefficients were calculated using Equation (1).

(1) $$r=\frac{\sum_{i=1}^{n}\left(X_{i}-\bar{X})(Y_{i}-\bar{Y}\right)}{\sqrt{\sum_{i=1}^{n}{\left(X_{i}-\bar{X}\right)}^{2}}\sqrt{\sum_{i=1}^{n}{\left(Y_{i}-\bar{Y}\right)}^{2}}}$$

**Figure 3:**
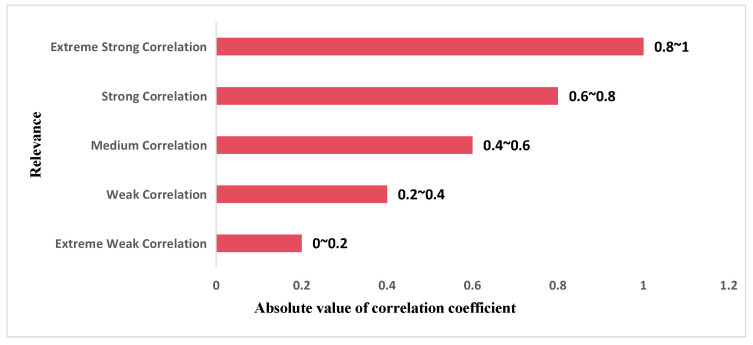
Relationship between absolute value of correlation coefficient and degree of correlation.

The correlation coefficient is a method for evaluating the correlation between two variables based on a monotonic function with a range of (−1, 1). The closer its absolute value is to 1, the stronger the correlation. The relationship between its value and degree of correlation is shown in Figure 3.

**Figure 4:**
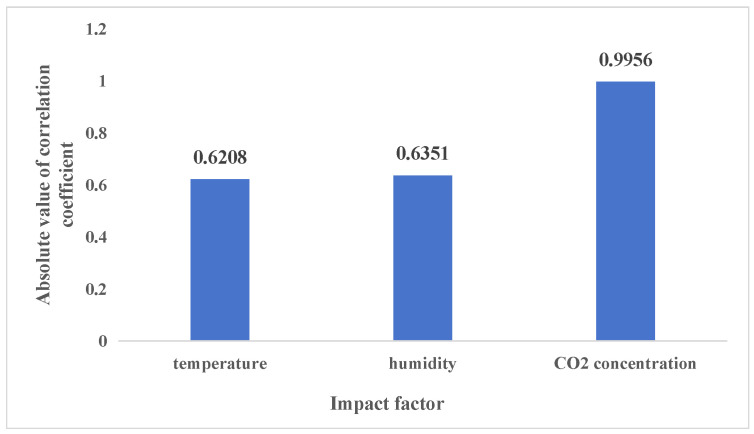
The correlation coefficient between the predicted value of carbon dioxide concentration and the historical values of the environmental parameters.

In this study, the correlation coefficient was used to estimate the correlation between the temperature, humidity, and CO_2_ concentration. The results are shown in Figure 4.

As shown in Figure 4, historical temperature and humidity were strongly correlated with the real-time CO_2_ concentration, and historical CO_2_ concentration was extremely strongly correlated with the real-time CO_2_ concentration.

This study only considered factors that were at least strongly correlated with the CO_2_ concentration. Therefore, temperature, humidity, and CO_2_ concentration were selected as inputs for the model.

### 2.3. LSTM

**Figure 5:**
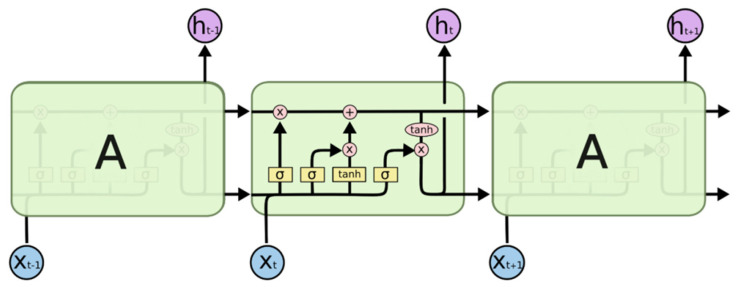
Graphical representation of the structure of LSTM.

LSTM is an RNN [18,19] with special structures that do not suffer from vanishing and exploding gradient problems during training with long sequences. In summary, LSTM performs better on longer sequences than regular RNNs do and is not affected by the gradient attenuation problems that occur with RNNs [13]. A graphical representation of the structure of LSTM is shown in Figure 5.

Memory, referred to as a cell that has the same shape as in the hidden state, is introduced into LSTM to record additional information. Input, output, and forget gates are included to control the memory [20].

#### 2.3.1. Forget Gate

A forget gate decides the types of information that should be discarded or retained. The information from both the previous hidden state and the current input is simultaneously transmitted to a sigmoid function. The output value is between zero and one. The information should be discarded or retained when the output value is closer to zero or one, respectively [21]. The forget gate is shown in Equation (2).

(2) $$f_{t}=\sigma (x_{t}w_{x}{}_{f}+h_{t-1}w_{hf}+b_{f})$$

#### 2.3.2. Input Gate

An input gate is used to update the state of the cell. First, the information of the previous hidden state, *h_t_*_−1_, and the information of the current input, *X_t_*, are transmitted to a sigmoid function [22]. The value is adjusted between zero and one to determine the types of information to be updated, with zero representing unimportant and one representing important information [23]. Second, *h_t_*_−1_ and *X_t_* are transmitted to a tanh function to create a vector with a new candidate value (candidate memory). Finally, the output value of the sigmoid function, It, is multiplied by the output value of the tanh function, which is the candidate memory [24]. The output value of the sigmoid function determines the types of information in the output value of the tanh function that are important and need to be retained [25]. The input gate is shown in Equation (3).

(3) $$i_{t}=\sigma (x_{t}w_{x}{}_{i}+h_{t-1}w_{hi}+b_{i})$$

#### 2.3.3. Output Gate

An output gate is used to determine the value of the next hidden state. A hidden state contains information from previous inputs [26]. First, *h_t_*_−1_ and *X_t_* are transmitted to a sigmoid function; second, the new cell state, *C_t_*, is transmitted to the tanh function [27]. Finally, the output of the tanh function is multiplied by the output of the sigmoid function, *O_t_*, to determine the types of information that the hidden state should carry, *h_t_* [28]. The hidden state is then used as the output of the current cell, transmitting *C_t_* and the new hidden state, *h_t_*_+1_, to the next time step [29]. The output gate is shown in Equation (4).

(4) $$o_{t}=\sigma (x_{t}w_{xo}+h_{t-1}w_{ho}+b_{i})$$

### 2.4. GRU

Gated recurrent neural networks are designed to better capture the dependencies of time series data with larger intervals. The GRU is a commonly used gated recurrent neural network [26].

In the hidden layers of RNNs, the gradients of variables may vanish or explode. Although gradient clipping can cope with the exploding gradient problem, it cannot resolve the vanishing gradient problem. The GRU, similar to LSTM, was developed to overcome problems such as the gradient in long-term memory and backpropagation [30].

The input and output structures of a GRU are the same as those of a regular RNN. The input includes both the input at moment *t*, *x_t_*, and the hidden layer state at moment *t* − 1, *h_t_*_−1_, which contain the information about the previous node. The output includes the output of the hidden node at moment *t*, *y_t_*, and the hidden state to be transmitted to the next node, *h_t_* [31].

**Figure 6:**
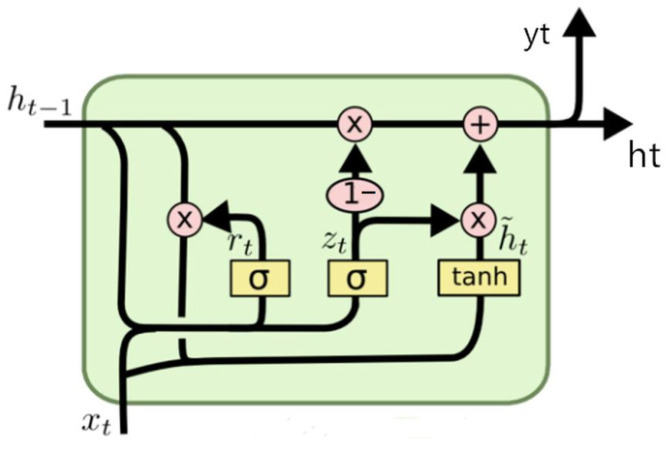
Graphical representation of the structure of a GRU.

Unlike LSTM, which has three gates, GRU has only two gates, which are the reset and update gates. The states of the two gates are obtained from the previous transmitted state, *h_t_*_−1_, and the input of the current node, *X_t_* [32]. A graphical representation of the structure of a GRU is shown in Figure 6.

#### 2.4.1. Update Gate

An update gate, *r_t_*, is used to control the extent to which the state information from the previous moment is applied in the current state, with more state information being used from the previous moment as the update gate value increases. The information from the previous and current moments is right-multiplied by a weight matrix. Subsequently, the summed data are fed into the update gate and multiplied by a sigmoid function to yield a value between zero and one [33]. The update gate is shown in Equations (5) and (6).

(5) $$r_{t}=\sigma (w_{r}\cdot [h_{t-1},x_{t}])$$

(6) $${\tilde{h}}_{t}=\tanh(w\cdot [r_{t}*h_{t-1},x_{t}])$$

#### 2.4.2. Reset Gate

A reset gate, *z_t_*, is used to control how much historical information from the previous state is written to the current candidate set, with less information from the previous state written as the reset gate value decreases [34]. The data processing of the reset gate is the same as that of the update gate, where the information from the previous and current moments is right-multiplied by a weight matrix. Subsequently, the summed data are fed into the reset gate and multiplied by a sigmoid function to yield a value between zero and one. The differences between the two weight matrices are their values and purposes [19]. The reset gate is shown in Equations (7) and (8).

(7) $$z_{t}=\sigma (w_{z}\cdot [h_{t-1},x_{t}])$$

(8) $$h_{t}=(1-z_{t})*h_{t-1}+z_{t}*{\tilde{h}}_{t}$$

### 2.5. Model Construction

(1) Hardware and software of the workstation

A workstation was set up for data processing, and the hardware configuration was as follows: CPU: AMD 7773X (AMD, Santa Clara, CA, USA), main board: GIGABYTE MZ72-HB0 (GIGABYTE, New Taipei City, Taiwan), graphics card: AMD RADEON PRO W7800 32 GB GDDR6 RDNA3 (AMD, Santa Clara, USA), ROM: 32 G DDR4 3200 RECC (KINGSTON, Fountain Valley, CA, USA), hard disk: SAMSUNG 1 TB M2 NVME (SAMSUNG, Seoul, Republic of Korea).

The software configuration was as follows, OS: Windows 10 Professional Edition (Microsoft, Redmond, WA, USA), Environmental configuration: Anaconda 3 and PyCharm Community Edition (Anaconda, Austin, TX, USA).

(2) Obtaining the datasets

**Figure 7:**
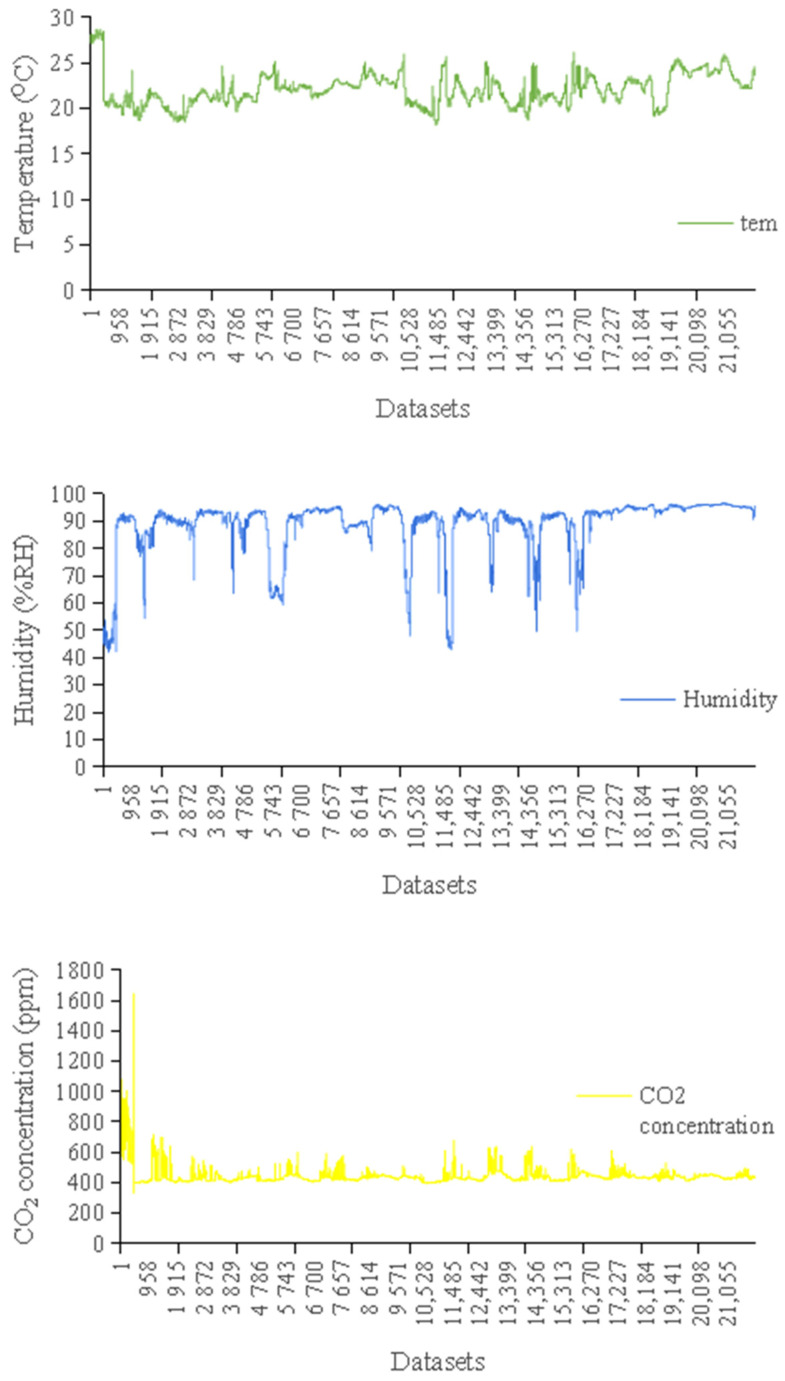
Time series diagrams of temperature, humidity, and CO_2_ concentration in the layer house.

The datasets for this study were obtained from an environmental monitoring system installed in the experimental layer house. The datasets took the average value of the measured data. There were 22,000 time series data in the datasets. The time series of the datasets is shown in Figure 7.

(3) Data pre-processing

**Figure 8:**
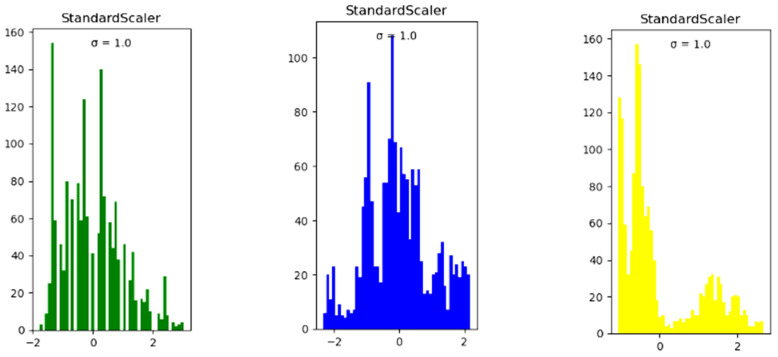
The standardized datasets of temperature, humidity, and CO_2_ concentration.

The sklearn standardization method was used to standardize all the feature data, as standardization can help prevent data with excessive bias from affecting the training results. The standardized datasets are shown in Figure 8.

(4) Time series: sliding window method

A convenient queue, deque, was used to specify the maximum queue length, maxlen, as 20, denoting that the time series length was 20. If queue length, deq, was more than 20, the first feature was deleted, whereas the 21st feature was appended to the 20th feature to constantly maintain a queue length of 20. As such, each time series had a shape of [5,13], denoting 20 rows of data and 5 columns of features.

After classifying the time series of all the data, the last 10 sets of the time series were deleted, as the last 10 rows of the feature data, sca_x, had no corresponding label value. Each series had a corresponding label, and the lengths of each series and its corresponding label were the same.

(5) Dataset partitioning

We obtained the processed time series and their corresponding labels. Subsequently, the training, validation, and test sets were proportionally divided. Overall, 80% of the datasets was used for training, 10% of the data was used for validation, and 10% of the data was used for testing. For the training set, shuffle(∙) was used to randomly disrupt the arrangement of the data rows to avoid contingency. The iterator, iter(∙), was set in conjunction with the next(∙) function to retrieve a batch of data from the training set.

(6) Construction of network models

We used a GRU network as an example here. The structure of the GRU model is shown in Table 1.

(7) Hyperparameters:

The configuration settings of the hyperparameters for the GRU and LSTM models is shown in Table 2.

(8) Hyperparameter optimization

This study applied the adaptive learning rate algorithm as Adam.

(9) Network training

The mean absolute error (MAE) between the predicted and label values was used as the loss function, and the mean squared logarithmic error (MSLE) was used as a metric for monitoring the networks. The MAE and MSLE were retained for each iteration during training. Early Stopping, a simple and effective method for avoiding overfitting, was used during model training. It works by monitoring the validation set performance during model training and stopping the training when the validation set performance is optimal, which can determine appropriate training rounds and avoid overfitting effectively.

(10) Inspecting training process information

For each iteration, the loss and monitoring metrics for both the training and validation sets were plotted.

(11) Forecasting

**Figure 9:**
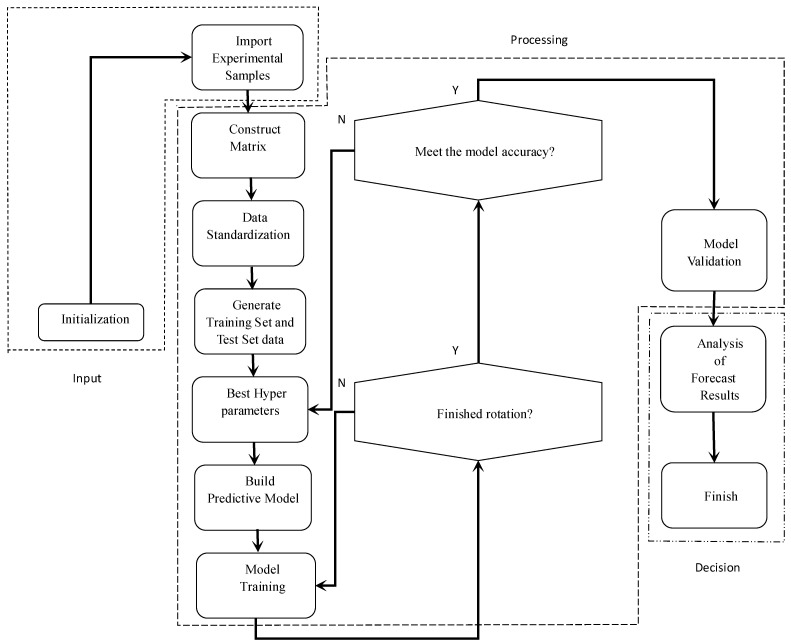
The experimental procedure of this study to develop the GRU and LSTM models.

The evaluate(∙) function was used to calculate the loss and monitoring metrics for the entire test set to obtain a timescale for each true value. The GRU and LSTM models’ prediction flow is shown in Figure 9.

## 3. Results

### 3.1. Evaluation Indicators

To evaluate the performance and assess the reliability of the models after training was completed, the input factors of the validation set were fed into the trained model to get the predicted values. These predictions were then compared to the actual observed values. Mean absolute error (MAE) between the predicted and label values was used as the loss function, and mean squared logarithmic error (MSLE) was used as a metric for monitoring the networks.

MSLE is the indicator used to evaluate the prediction model. It is the average value of logarithmic error, which can be used to measure the prediction ability of the model for data with large values. MSLE is calculated as shown in Equation (9).

(9) $$\mathrm{MSLE}=\frac{1}{N}\sum [\log(y_{i}+1)-\log({\hat{y}}_{i}+1){]}^{2}$$

The range of MAE is (0,+∞), with 0 indicating a perfect model where the predicted value matches the true value exactly. The larger the error, the larger the MAE value. MAE is shown in Equation (10).

(10) $$\mathrm{MAE}=\frac{1}{N}\sum_{i=1}^{N}\left|{\hat{y}}_{i}-y_{i}\right|$$

### 3.2. Predictive Evaluation

**Figure 10:**
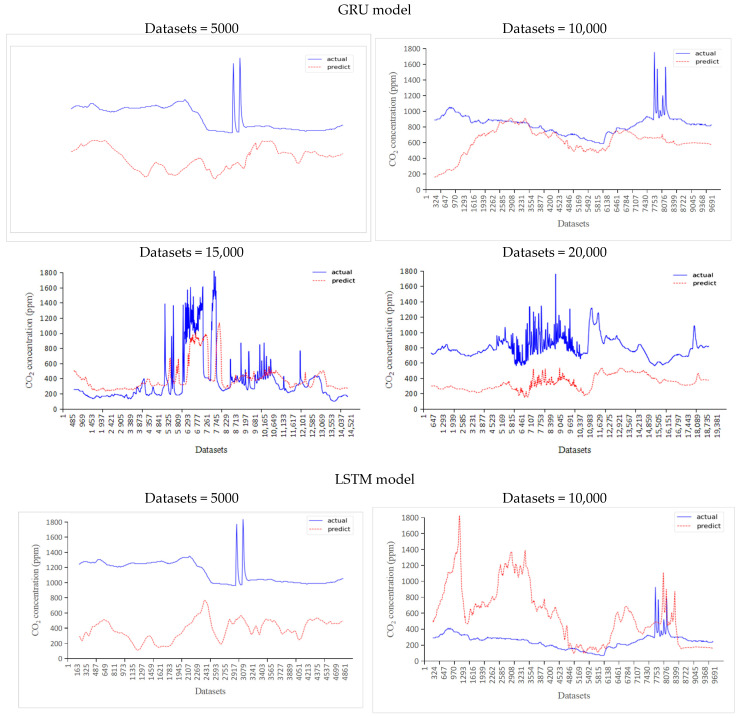

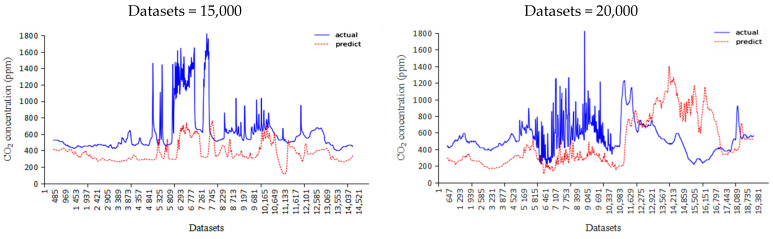
Comparison of predicted and actual values with the GRU and LSTM models.

The sample sizes of the test sets were 5000–20,000, and the predicted and actual values are shown in Figure 10.

It can be seen from Figure 10 that when the number of datasets put into the GRU model was increased from 5000 to 15,000, the agreement between the predicted and actual values gradually increased. When 20,000 datasets were put into the GRU model, the degree of agreement between the predicted and actual values slightly decreased. We concluded that when the number of datasets put into the GRU model was within an appropriate range, the predication accuracy increased with the number of datasets.

When the number of datasets input to the LSTM model was increased from 5000 to 20,000, the agreement between the predicted and actual values gradually increased.

### 3.3. Evaluation of Model Performance by Plotting Training and Validation Loss Curves

The training and validation loss curves are two evaluation metrics commonly used in the deep learning training process to represent the errors of a model during training and validation.

#### 3.3.1. The Train_Loss and Train_Msle of the GRU Model and LSTM Model

Training loss refers to the model’s error on the training set, indicating the magnitude of the model’s prediction error on the training samples during the training process.

**Figure 11:**
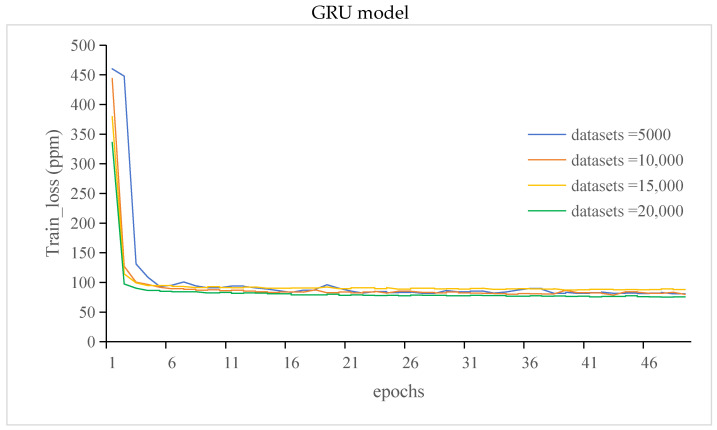

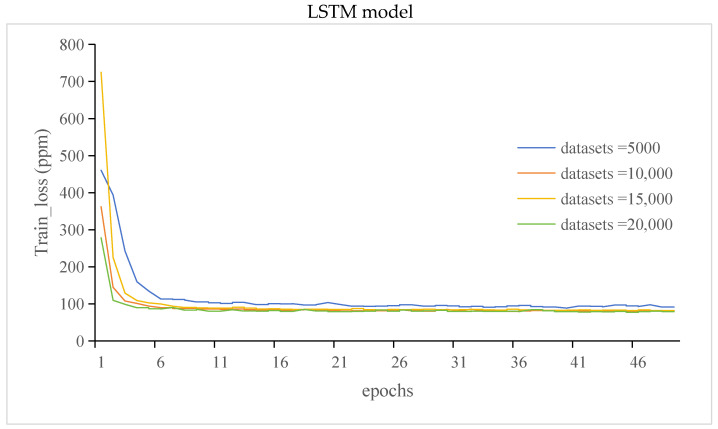
The train_loss of the GRU and LSTM models.

The train_loss of the GRU and LSTM models is shown in Figure 11.

It can be seen from Figure 11 that when 5000 datasets were put into the models, the training_loss curve of the GRU model showed that the training error stayed at the maximum for one training cycle, whereas the curve of the LSTM model rapidly decreased after training started. During model training, the validation set was used to test the performance of the models at regular intervals to obtain the val_loss results.

The training_loss of the GRU and LSTM models gradually decreased as the number of training sessions gradually increased. The models produced more accurate predictions on the training samples because they had learned more features and patterns. Therefore, the training_loss curve showed a decreasing trend as the number of training sessions increased.

**Figure 12:**
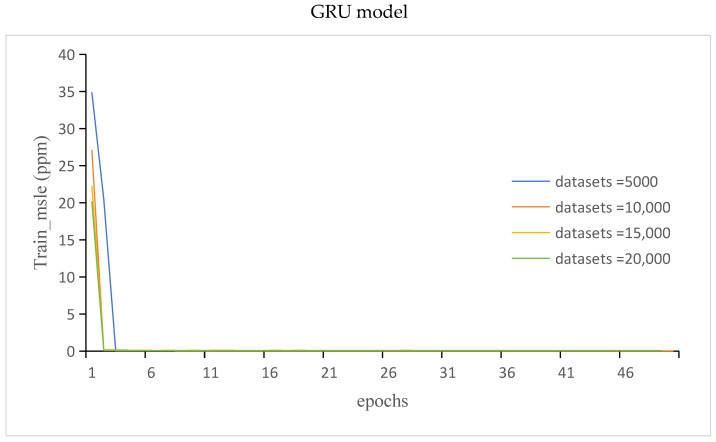

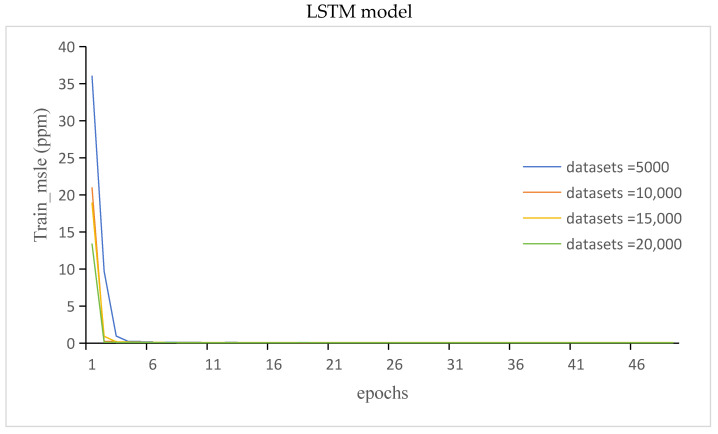
The train_msle of the GRU and LSTM models.

The train_msle of the GRU and LSTM models is shown in Figure 12.

#### 3.3.2. The Val_Loss and Val_Msle of GRU Model and LSTM Model

Validation loss refers to the model’s error on the validation set, indicating the magnitude of the model’s prediction error on the unknown data during the training process. The validation set accounts for a portion of the data randomly cleaned from the training set, which includes the data that the models had not yet encountered during the training process. The validation set is used to evaluate the ability of a model to predict unknown data.

**Figure 13:**
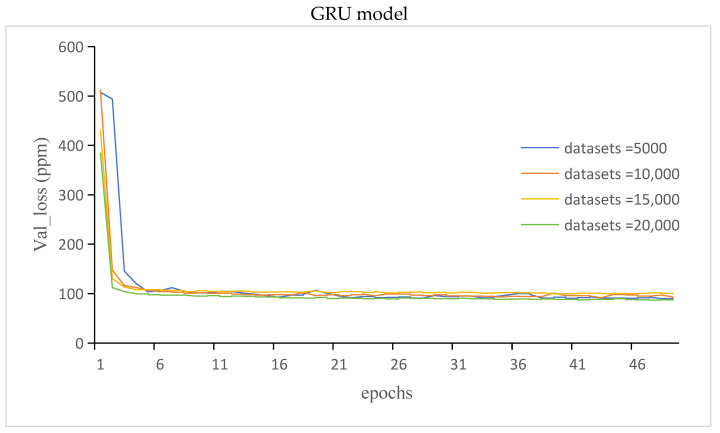

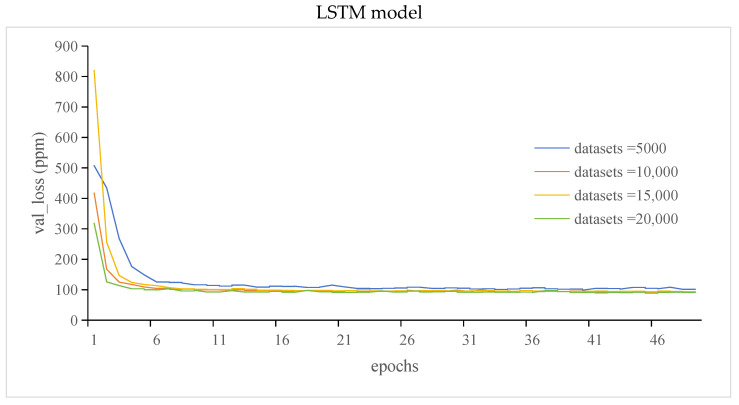
The val_loss of the GRU and LSTM models.

The val_loss of the GRU and LSTM models is shown in Figure 13.

**Figure 14:**
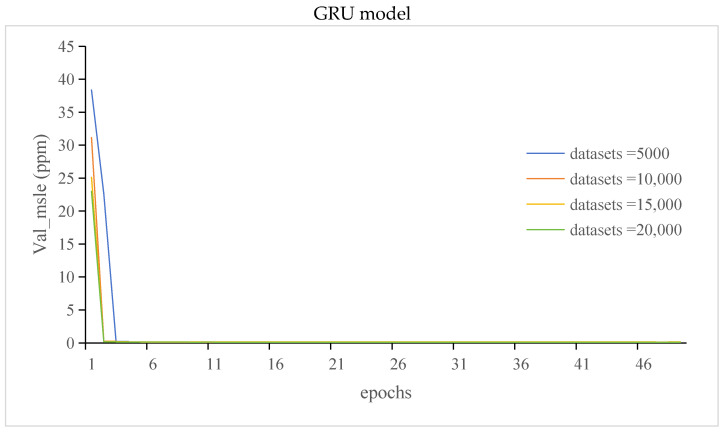

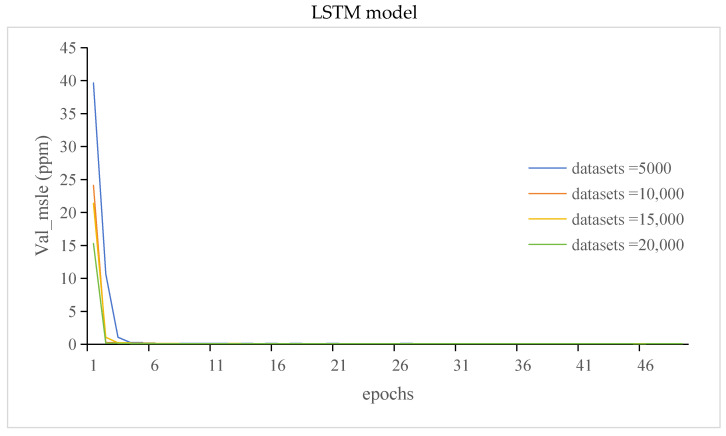
The val_msle of the GRU and LSTM models.

The val_msle of the GRU and LSTM models is shown in Figure 14.

#### 3.3.3. Overfitting Check by Cross-Validation

**Figure 15:**
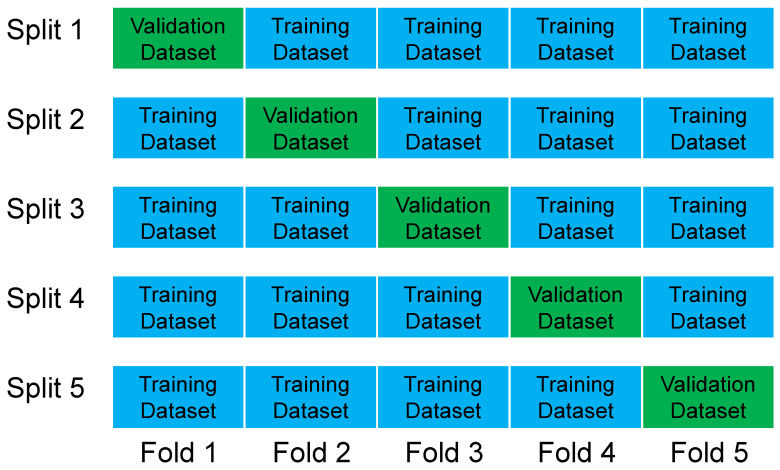
Five-Fold cross-validation to check for overfitting.

This study adopted Five-Fold cross-validation. We divided the datasets into five non-overlapping sub-datasets and then performed five model trainings and validations. Every time, we validated the model using one large dataset and trained the model using the other four large datasets. In these five training and validation sessions, the datasets used to validate the model were different each time. Finally, we calculated the averages of training loss and validation loss for these five iterations. The Five-Fold cross-validation is shown in Figure 15.

A comparison was made between the train_loss and val_loss of the LSTM model and the GRU model to determine if there was overfitting in the model. The comparison results are shown in Table 3.

It can be seen from Table 3 that when the train_loss of the LSTM model and GRU decreased, val_loss also decreased, and there was no overfitting in the model.

#### 3.3.4. Model Application

A comparative field test for the prediction model was conducted in an experimental layer house, in where the actual CO_2_ concentration in the layer house was used as the model input to predict the CO_2_ concentration after one minute.

The predicted and actual values of the CO_2_ concentration were considered as the basis for the ventilation of the layer house, and the upper limit of CO_2_ concentration was set as 500 ppm.

If the predicted value of the CO_2_ concentration was used as the basis for ventilation, the fan was turned on when the predicted value reached 500 ppm.

If the actual value of the CO_2_ concentration was used as the basis for ventilation, the fan was turned on when the actual value reached 500 ppm.

The CO_2_ concentration and fan status of the layer house in both cases are shown in Table 4.

Table 4 shows that if the predicted value was used as the basis, at 9:11:40, the predicted and actual values were 500.0410 ppm and 499.9687, respectively, and the fan was turned on. At 09:11:55, the actual value was 499.2547 ppm, and the fan was turned off.

If the actual value was used as the basis, when the actual value was 500.1364 ppm, the fan was turned on. At 09:12:00, the actual CO_2_ concentration was 499.3159 ppm, and the fan was turned off.

It could be judged from the experimental results that if the predicted value was used as the basis for ventilation, the CO_2_ concentration in the layer house could be ensured to remain below the limit value at all times, and the total operation time of the fan was 15 s.

If the actual value was used as the basis for ventilation, the CO_2_ concentration exceeded the limit value for 10 s after starting the fan, and the total operation time of the fan was 20 s.

This concludes that using the predicted value as the basis for adjusting the operating status of fans could reduce the CO_2_ concentration faster and ensure the constraints of the CO_2_ concentration in the chicken house.

## 4. Discussion

It can be seen from Figure 10 that when the number of datasets of the GRU and LSTM models gradually increased, the agreement of the predicted and actual values gradually increased.

However, when the number of datasets reached 20,000, the agreement of the predicted and actual values of the GRU model decreased, while the agreement of the LSTM model did not decrease.

It could be concluded that the model had optimal datasets. When the number of datasets was within the optimal range, the agreement of the predicted and actual values increased with number of datasets. When the number of datasets exceeded the optimal range, the agreement of the predicted and actual values was uncertain.

In order to acquire the optimal number of datasets, prediction was attempted with 15,000 to 20,000 datasets, with 1000 datasets added each time.

**Figure 16:**
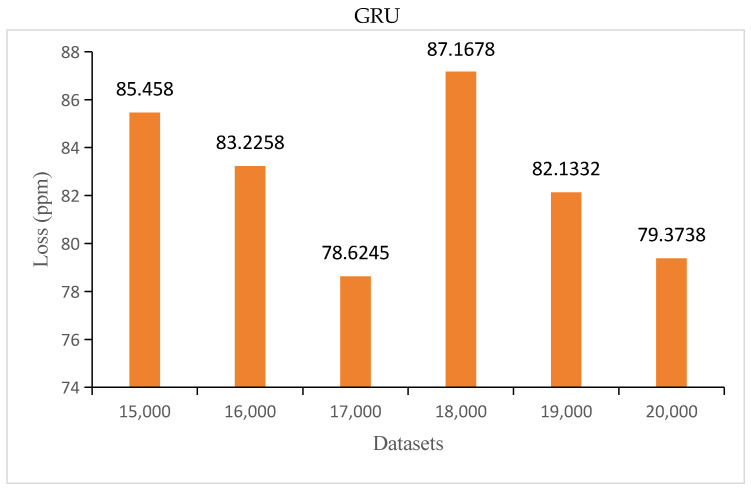

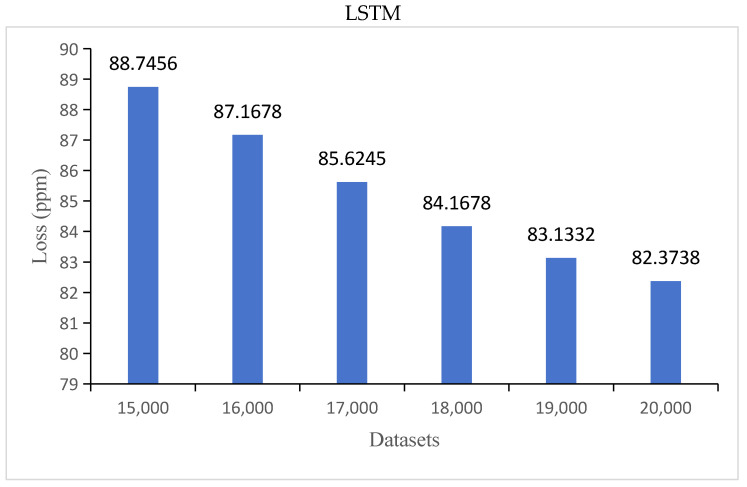
Loss of the GRU and LSTM models within 15,000 to 20,000 datasets.

The MAE of the GRU and LSTM is shown in Figure 16.

It can be seen from Figure 16 that when the number of datasets was between 15,000 and 17,000, the prediction loss of the GRU model decreased with the increase of the number of datasets. When the number of datasets exceeded 17,000, the agreement between the predicted and actual values was uncertain. It could be concluded that the optimal number of datasets for the GRU model was between 15,000 and 17,000. When the number of datasets was between 15,000 and 20,000, the prediction loss of the LSTM model decreased with increasing number of datasets, and the agreement between the predicted and real values was certain. It could be concluded that the optimal number of datasets for the LSTM model was between 15,000 and 20,000.

It can be seen from Figure 11, Figure 12, Figure 13 and Figure 14 that compared with those of the GRU model, the LSTM model has more stable but lower prediction speed and more loss. The reason was that the two models had different structures. The LSTM model contained three gates the, forget gate, the input gate, and the output gate, and the LSTM model had more parameters. The GRU model contained two gates, the update gate and the reset gate. Due to the structure of the model, the stability of the LSTM model was higher than that of the GRU model, and the prediction accuracy and speed were lower than those of the GRU model.

The loss of the GRU model was 70.8077 to 126.7029 ppm, and the loss of LSTM model was 78.8596 to 136.0896 ppm. The prediction time of the GRU model was 16 to 24 ms, and the prediction time of the LSTM model was 17 to 26 ms.

When the model was applied in a layer house, the measured value of the carbon dioxide concentration in the house was used as the input, which required high accuracy of the layer house carbon dioxide measurement device. The layer house manager needs to invest in hardware procurement costs when using this model, which is applicable to managers of large-scale farming.

## 5. Conclusions

(1) In this study, an extensive examination of the performance of GRU and LSTM models in predicting environmental parameters, specifically the CO_2_ concentration in a layer house, provided significant insights and contributions to the field of predictive modeling for agricultural and environmental applications.
(2) According to the correlation coefficients, the layer house temperature, humidity, and CO_2_ concentration were selected as the feature data, and a model for predicting the CO_2_ concentration in a layer house was constructed based on the GRU and LSTM.
(3) Different datasets were selected, and the corresponding prediction results were obtained. The training and validation errors of the GRU and LSTM models were analyzed. The results showed that there was an optimal range of the number of datasets for the prediction model, and the loss of the model was minimal within this range
(4) MAE between the predicted and label values was used as the loss function, and MSLE was used as a metric for monitoring the networks. MAE and MSLE were retained for each iteration during training. For each iteration, the loss and monitoring metrics of both the training set and validation set were plotted. The evaluate(∙) function was used to calculate the loss and monitoring metrics for the entire test set to obtain a timescale for each true value.
(5) While increasing the dataset size yielded an improvement in prediction accuracy for both GRU and LSTM models, the findings noted a decline in prediction accuracy for the GRU model when 20,000 datasets were utilized. This suggests that the GRU model’s performance might plateau or decline beyond a certain dataset threshold, indicating a limitation in handling larger datasets. We will focus on solving this problem in future work.
(6) The datasets for this study were collected between June and July 2023. In future research, the collection time of the datasets will be extended to four seasons to improve the applicability and robustness of the model.
(7) This study will address the computational efficiency issue of the model in future work.

## Figures and Tables

**Table 1:** The structure of the GRU model.

Model: “GRU”		
Layer (type)	Output Shape	Parameter
input_4 (InputLayer)	[(None, 20, 5)]	0
gru_3 (GRU)	(None, 20, 8)	360
dropout_12 (Dropout)	(None, 20, 8)	0
gru_4 (GRU)	(None, 20, 16)	1248
dropout_13 (Dropout)	(None, 20, 16)	0
gru_5 (GRU)	(None, 32)	4800
dropout_14 (Dropout)	(None, 32)	0
dense_6 (Dense)	(None, 16)	528
dropout_15 (Dropout)	(None, 16)	0
dense_7 (Dense)	(None, 1)	17
Total parameters: 6953		
Trainable parameters: 6953		
Non-trainable parameters: 0		

**Table 2:** Hyperparameters for the GRU and LSTM models.

Hyperparameters	Value
timestep	1
batch_size	32
feature_size	1
hidden_size	256
output_size	1
num_layers	2
epochs	100
best_loss	0
learning_rate	0.0003

**Table 3:** Comparison of training error and testing error.

Model	Datasets	Train_Loss	Val_Loss
LSTM	5000	90.8156	91.6452
	10,000	88.0604	89.8628
	15,000	86.0255	87.3469
	20,000	84.8316	85.3657
GRU	5000	80.7534	82.4733
	10,000	78.8040	80.3956
	15,000	76.4618	78.3562
	20,000	79.3265	80.6643

**Table 4:** CO_2_ concentration and fan operation state inlayer house under different ventilation mechanism.

Fan Opening Basis	Actual Value and Measuring Time of Carbon Dioxide Concentration in Layer House	Predict Value and Corresponding Time of Carbon Dioxide Concentration in Layer House	Status of Fan
Predicted value of carbon dioxide concentration	495.8376 ppm 09:10:25	499.2168 ppm 09:11:25	Off
	495.3592 ppm 09:10:40	500.0410 ppm 09:11:40	Off
	499.9687 ppm 09:11:40	503.6828 ppm 09:12:40	On
	499.2547 ppm 09:11:55	495.3158 ppm 09:12:55	Off
Actual value of carbon dioxide concentration	499.6548 ppm 09:10:40		Off
	500.1364 ppm 09:11:40		On
	500.0129 ppm 09:11:50		On
	499.3159 ppm 09:12:00		Off

## Data Availability

The data presented in this study are available on request from the corresponding author.
